# Increased risks of cardiovascular diseases and insulin resistance among the Orang Asli in Peninsular Malaysia

**DOI:** 10.1186/s12889-016-2848-9

**Published:** 2016-03-24

**Authors:** Tuan Azlin Tuan Abdul Aziz, Lay Kek Teh, Muhd Hanis Md Idris, Zakaria Bannur, Lydiatul Shima Ashari, Adzrool Idzwan Ismail, Aminuddin Ahmad, Kamarudzaman Md Isa, Fadzilah Mohd Nor, Thuhairah Hasrah Abdul Rahman, Syahrul Azlin Binti Shaari, Hamid Jan Jan Mohamed, Nornazliya Mohamad, Mohd Zaki Salleh

**Affiliations:** Integrative Pharmacogenomics Institute (iPROMISE), Universiti Teknologi MARA (UiTM), Selangor Campus, Selangor, Malaysia; Faculty of Pharmacy, Universiti Teknologi MARA (UiTM), Selangor Campus, Selangor, Malaysia; Nutrition Programme, School of Health Sciences, Universiti Sains Malaysia, Kelantan, Malaysia; Faculty of Art and Design, Universiti Teknologi MARA (UiTM), Shah Alam, Selangor Malaysia; Faculty of Medicine, Universiti Teknologi MARA (UiTM), Sungai Buloh, Selangor Malaysia

**Keywords:** Biochemical profiles, Health status, Liver functions, Lipid profiles, Orang Asli, Framingham Risk Score, Insulin resistance

## Abstract

**Background:**

Despite the strategic development plan by the authorities for the Orang Asli, there are six subtribes of which their population numbers are small (less than 700). These minorities were not included in most of the health related studies published thus far. A comprehensive physiological and biomedical updates on these small subtribes in comparison to the larger subtribes and the urban Malay population is timely and important to help provide appropriate measures to prevent further reduction in the numbers of the Orang Asli.

**Methods:**

A total of 191 Orang Asli from different villages in Peninsular Malaysia and 115 healthy urban Malays were recruited. Medical examinations and biochemical analyses were conducted. Framingham risk scores were determined. Data was analyzed using IBM SPSS Statistics, Version 20.0.

**Results:**

A higher percentage of the Orang Asli showed high insulin levels and hsCRP compared to the healthy Malays denoting possible risk of insulin resistance. High incidences of low HDL-c levels were observed in all the Orang Asli from the six subtribes but none was detected among the urban Malays. A higher percentage of inlanders (21.1 % of the males and 4.2 % of the females) were categorized to have high Framingham Risk Score.

**Conclusions:**

Orang Asli staying both in the inlands and peripheries are predisposed to cardiovascular diseases and insulin resistance diabetes mellitus. The perception of Orang Asli being healthier than the urban people no longer holds. We believed that this information is important to the relevant parties in strategizing a healthier community of the Orang Asli to avoid the vanishing of the vulnerable group(s).

## Background

The aborigines are indigenous people whom have been the subjects for studies to track migration patterns of the modern human. They are also the focus of research to understand the mechanisms of evolution and natural selection; and the differences they carry may shed lights towards the evolution of diseases and mechanisms.

*‘Orang Asli’* is known as the ‘first people’ or ‘original people’ in Peninsular Malaysia [1]. The Orang Asli was categorised into three tribes namely the Proto-Malay, Senoi and Negrito; and each tribe was further grouped into smaller sub-tribes. They comprised of 0.6 % of the Malaysian population. In 2008, the total population of Orang Asli in Malaysia was estimated to be 178,163. Senoi has a population of 97,732 and represents the largest population of the Orang Asli. The second largest population is Proto-Malay (population of 67,605) and followed by the Negrito which comprised of 5,599 individuals [2].

Negrito is known to be the earliest Orang Asli arrived in Peninsular Malaysia. It was thought that they arrived about 25,000 years ago [3] but other theory suggested that they may had arrived earlier, about 60,000 years ago [4]. The Negrito also has the smallest population size among the three tribes of Orang Asli where the settlements are isolated and scattered but mainly distributed in the Northern and middle part of the Peninsular Malaysia [5]. Senoi resides mainly in the middle to northern part of Peninsular Malaysia. Senoi was thought to reach Peninsular Malaysia via the second wave of migration from the mountain areas of Cambodia and Vietnam [6]. Senoi is divided into six subtribes comprising the Semai, Temiar, Che Wong, Jah Hut, Semoq Beri and Mah Meri [1]. The Proto Malay also known as the Aboriginal Malay [7], is the second largest group of Orang Asli which are categorised into six sub-tribes; Jakun/Orang Hulu, Temuan, Semelai, Orang Kuala, Orang Kanaq and Seletar. According to Fix [7], Proto Malay migrated from the middle part of Asia (Yunnan) and travelled through Indo-China before they arrived in Peninsular Malaysia in 2,000 B.C. They were seafaring people and resided mostly in the central and southern regions of Peninsular Malaysia [3, 8].

Since 1957, the Government of Malaysia as well as other non-government agencies have embarked on different development programmes to bring advancement to the quality of life of the communities of Orang Asli. The programmes include educational, financial, agricultural and livestock subsidies, health services, free food, and housing opportunities [9, 10]. About one third of the Orang Asli are still staying in the inlands (37 % in the remote areas) while others have been relocated to new resettlement area such as the outskirt of existing rural villages (61 %) and vicinity of existing townships (2 %) [11]. Despite the planned development, most of the Orang Asli in the country are plagued with negative pressures in both socio-economics and health aspects. The factors that contribute to this observation are continuously under research by many in order to help find solutions to improve the situation.

The Orang Asli was reported to have a lower health status compared to the other ethnic groups in Malaysia [12]. According to Rusaslina [13], the average life expectancy of the Orang Asli was 53 years; 54 years for females and 52 years for males as compared to 72 and 68 years for females and males respectively for the other major ethnic groups in Malaysia. The females of the Orang Asli experienced a lower life expectancy at birth probably which was due to their higher maternal death rates resulting from childbirth or poor maternal health [14, 15]. One of the reasons that contributed to high mortality was the lack of access to the modern medical facilities; the other reason was the strong beliefs that their lives were controlled by supernatural beings and thirdly was the lack of trust in modern medicine. Genetic vulnerability, socio-economic disadvantages, resource alienation and political oppression were the other culprits in causing the low quality of life [16]. Hayati et al. [17] reported issues on under-nutrition and socio-demographic characteristics with the health status below the satisfactory level among the Orang Asli. Poor hygiene had exposed them to several infections such as soil-transmitted helminths [18], intestinal parasitic infection [19], and malaria [20].

Studies thus far had provided snap shots of the health status of a few subtribes of the Orang Asli especially on those with larger population size. In this study, we report for the first time, the medical information of several sub-tribes of the Orang Asli which are being categorised as fragile due to their small population size. This created research interest to investigate factors that caused the small numbers of these subtribes. After a review on the population size, the subtribes with small population size (n < 700) include Che Wong (*n* = 651), Kanaq (*n* = 148) and Kensiu (*n* = 327). Coincidentally, these sub-tribes have been relocated under the resettlement programmes to the peripheries of towns from the inlands. On the other hand, the Semai has the largest population size number (*n* = 51,313) and the majority of them are still living in the highland and are adapted to activities based on forests resources such as hunting, fishing, gathering and engaging in swidden farming. The subtribes of Lanoh and Bateq still live in the inlands and some still prefer the nomadic life style despite the infrastructures provided by the Government. We therefore, investigated the health status of the Orang Asli in the inlands and peripheries where the inlanders still stay close to the original habitat or remote areas of the jungles while those at the peripheries are those who had undergone resettlement. Therefore, the Orang Asli in the inlands are believed to be healthier than those living at the peripheries of town. We aimed to investigate the effects of resettlement on the changes of life styles and health status of the Orang Asli. A group of healthy Malays were used as controls as they live in towns in the same geographical areas as the Orang Asli.

## Methods

### Subjects and sampling

**Fig. 1:**
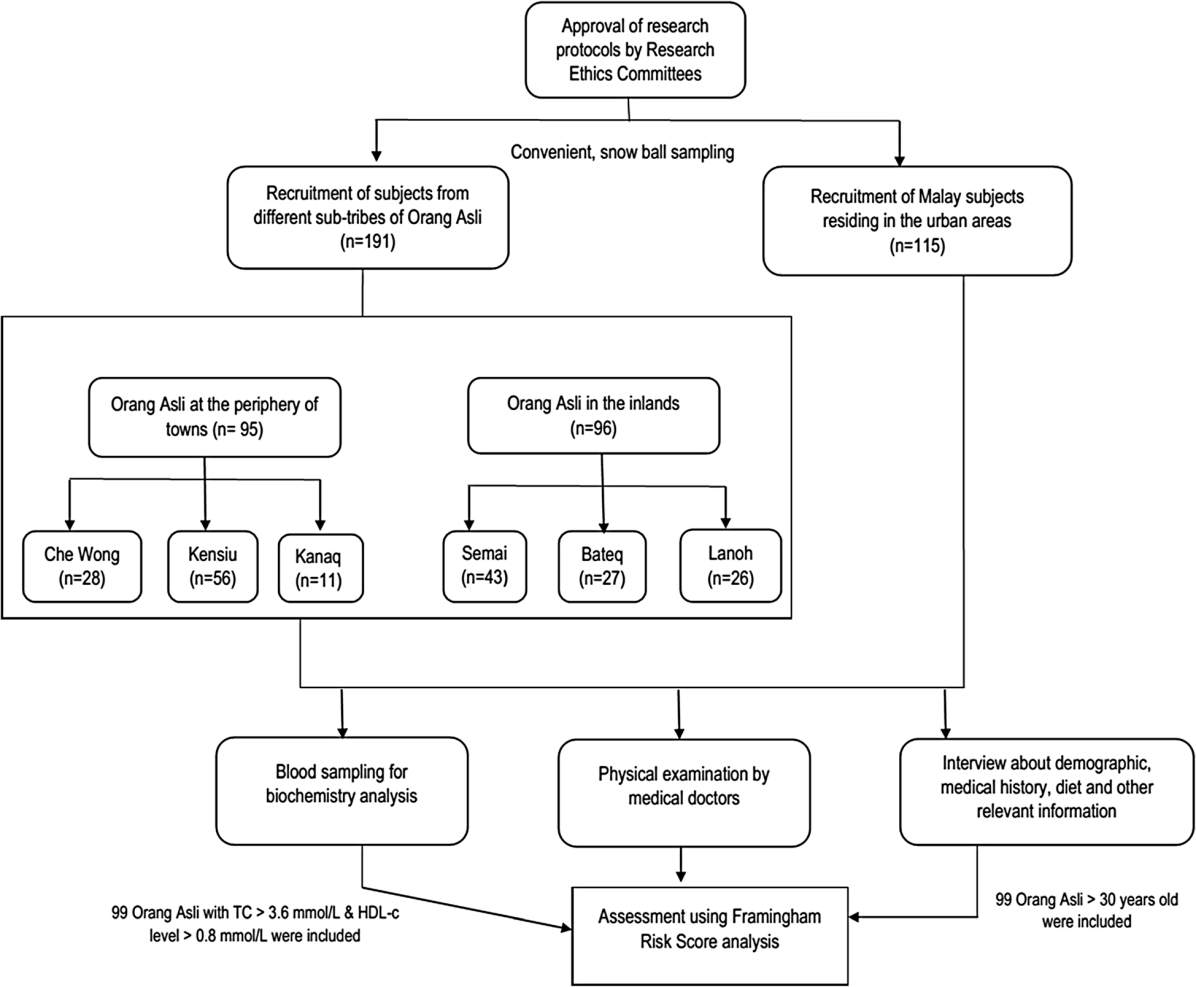
Study design

This study was approved by the Human Research Ethics Committee, Universiti Teknologi MRA (600-RMI (51/6/01)). The protocol of the study strictly adhered to the guide to protect the interest of the Orang Asli as determined by the Department of Orang Asli, Malaysia (JAKOA. PP. 30. 052 Jld.5 (62)). The overall study design is shown in Fig. 1.

**Fig. 2:**
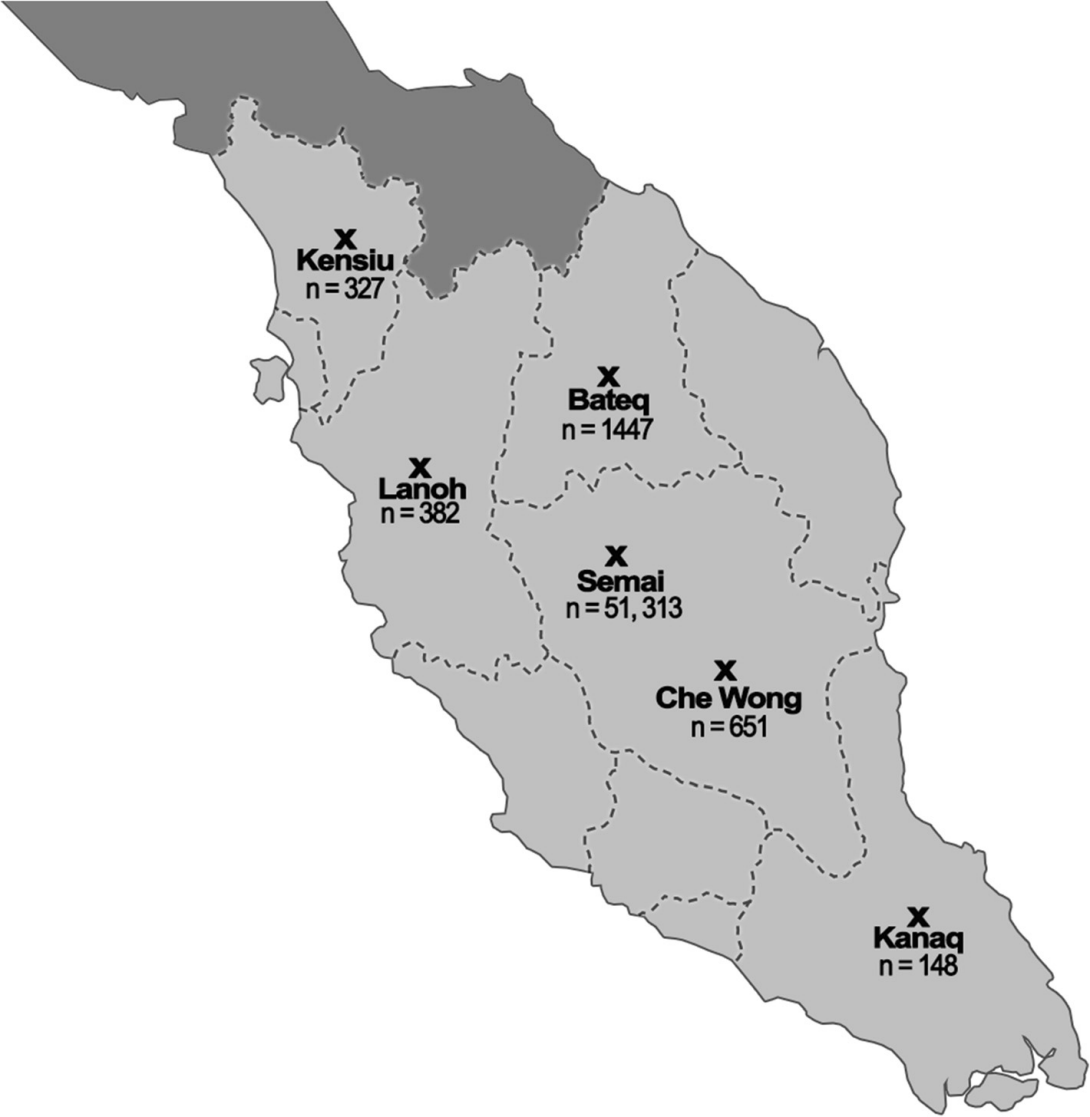
Location and population size of Orang Asli participated in the study

Convenient, snow ball approach was used in the sampling of the subjects in this study. A total of 191 blood samples were obtained from the six subtribes of the Orang Asli representing those staying inland (96 subjects) and periphery of towns (95 subjects). All the samples were collected from various villages in 7 different states in Peninsular Malaysia. Three of the subtribes namely Kanaq, Kensiu and Che Wong had been relocated to the periphery of towns and have population sizes which are less than 700 each. The map describing the locations and the population size of the sub-tribes is shown in Fig. 2. The other three sub-tribes, Semai, Bateq and Lanoh are inlanders with bigger population sizes of thousands. Adults, age above 18 years old, self-claimed healthy unrelated individuals were recruited. In addition, 115 healthy Malays living in the same geographical areas were also recruited as the urban control group in order to investigate the effect of resettlement. All the subjects were recruited after detailed explanation of the objectives and protocols of the study and written informed consents were obtained. The information on the subjects were ascertained of confidentiality and publications of the data in all cases will not allow identification of any specific individual.

### Inclusion and exclusion criteria

The inclusion criteria for the subjects were ability to provide written informed consent for the participation and information on ethnicity; as well as have no known systemic disease. The subjects were excluded if they were not clear with the protocols of the study, did not consent to participate in the study, bedridden, were mentally or physically disabled, pregnant or less than 6 months post-delivery. Those with known systemic disease as well as unsure of their own ethnicity were excluded.

### Biochemical analysis

Overnight fasting venous blood samples were drawn by medical doctors for determination of total cholesterol, high density lipoprotein cholesterol (HDL-c), low density lipoprotein cholesterol (LDL-c), triglycerides (TG), urea, creatinine, sodium, potassium, albumin, alkaline phosphatase, alanaine transaminase, gamma-glutamyltransferase, total protein, fasting plasma glucose, direct bilirubin, bilirubin total, insulin, ferritin, glycated haemoglobin (HbA1c) as well as high-sensitive C-reactive protein (hs-CRP). Biochemical analysis was performed at the Centre for Pathology Diagnostic and Research Laboratories (CPDRL- compliant to ISO15819), Universiti Teknologi MARA (UiTM), Sungai Buloh. The biochemical profiles were analysed using COBAS INTEGRA 800 analyzer with appropriate quality controls according to the particular test parameters (according to the manufacturer’s instructions). The inter assay coefficient of variants (CV) for each assay are shown in Tables 1 and 2.

**Table 1:** General characteristic of the Orang Asli and Malays that particiated in this study

Characteristics	Orang Asli		Malays	Total, n (%)
	Inland, n (%)	Periphery, n (%)	Urban, n (%)	
Overall	96 (31.4)	95 (31.0)	115 (37.6)	306 (100)
Age groups (years)				
15–18	5 (1.70)	8 (2.7)	12 (4.0)	25 (8.4)
19–29	38 (12.8)	42 (14.1)	66 (22.2)	146 (49.2)
30–49	36 (12.1)	28 (9.4)	33 (11.1)	97 (32.7)
50–59	9 (3.0)	11 (3.7)	1 (0.3)	21 (7.1)
>60	5 (1.7)	3 (1.0)	0	8 (2.7)
Gender				
Male	57 (51.4)	28 (25.2)	26 (23.4)	111 (36.3)
Female	39 (20.0)	67 (34.4)	89 (45.6)	195 (63.7)
Tribes				
Semai	43 (14.1)	0	-	43 (14.1)
Bateq	27 (8.8)	0	-	26 (8.8)
Che Wong	0	28 (9.2)	-	28 (9.2)
Kanaq	0	11 (3.6)	-	11 (3.6)
Kensiu	0	56 (18.3)	-	56 (18.3)
Lanoh	26 (8.5)	0	-	26 (8.5)

**Table 2:** Comparison of biochemical profiles among the male Orang Asli and male Urban Malays

Biochemical profiles	Asian Reference range	Orang Asli (male)							CV
		Inland			Periphery				
		Semai (*n* = 19)	Bateq (*n* = 23)	Lanoh (*n* = ^15)^	Kanaq (*n* = 2)	Kensiu (*n* = 17)	Che Wong (*n* = 9)	Malay (*n* = 26)	
Lipid profile									
TC, mmol/L^a^	Mean (SD)	3.95 (0.96)	4.09 (0.73)	5.13 (1.12)^c,d^	3.45 (0.21)	4.59 (0.90)	5.01 (1.22)	5.59 (0.88)^c,d,g,h^	0.81
% of the subjects	>5.2	5.26	4.35	46.67	0	35.29	55.56	69.23	
HDL-C, mmol/L^a^	Mean (SD)	0.99 (0.18)	1.14 (0.23)	1.18 (0.37)	0.70 (0.00)^c,d,h,i^	1.18 (0.45)	1.00 (0.51)	1.48 (0.29)^c,d,g^	1.13
% of the subjects	<1.0	31.50	21.74	40.00	100	29.41	55.56	0	
LDL-C, mmol/L^a^	Mean (SD)	2.34 (0.85)	2.52 (0.63)	3.25 (0.90)^c^	2.15 (0.35)	2.88 (0.95)	3.29 (1.19)	3.67 (0.81)^c,d^	1.10
% of the subjects	>4.1 (very high risk)	0	0	20.00	0	11.76	22.22	34.62	
	<4.1 (high risk)	15.79	8.70	13.33	0	23.53	33.33	23.08	
	<3.4 (moderate)	21.05	39.13	46.67	0	29.41	33.33	34.62	
	<2.6 (low risk)	63.16	52.17	20.00	100	35.29	11.11	7.69	
TG, mmol/L^a^	Mean (SD)	1.36 (0.96)	0.91 (0.36)	1.61 (0.82)^d^	1.15 (0.21)	1.17 (0.50)	1.59 (0.82)	1.00 (0.34)	1.60
% of the subjects	>1.7	26.32	8.70	33.33	0	11.76	33.33	7.69	
Endocrine tests									
FBG, mmol/L^b^	Mean (SD)	5.05 (1.00)	5.10 (0.80)	5.70 (3.00)^c,d^	5.85 (0.00)	5.80 (2.40)^c^	5.30 (0.80)	4.90 (0.50)^h,i^	0.38
% of the subjects	3.5–6.0 (normal)	88.89	86.36	50.00	100	53.33	88.89	96.00	
	6.1–6.9 (IFG)	11.11	9.09	21.43	0	20.00	11.11	4.00	
	>7.0 (DM)	0	4.55	28.57	0	26.67	0	0	
HbAlc, %^b^	Mean (SD)	5.20 (0.50)	5.30 (0.40)	5.45 (0.60)	-	5.2	5.20 (0.60)	5.40 (0.40)	0.80
% of the subjects	<4.8	5.26	0	7.14	-	0	11.11	11.54	
	4.8–5.9	94.74	100.00	78.57	-	100.00	88.89	88.46	
	>5.9	0	0	14.29	-	0	0	0	
Insulin, pmol/L^a^	Mean (SD)	91.57 (86.82)	95.89 (88.44)	176.95 (220.37)	17.7 (4.67)^e,i^	98.06 (102.28)	140.88 (111.56)	66.54 (83.29)	1.70
% of the subjects	<17.8	15.79	0	0	50.00	5.88	0	3.85	
	17.8–173.0	68.42	73.91	80.00	50.00	82.35	55.56	92.31	
	>173.0	15.79	26.09	20.00	0	11.76	44.44	3.85	
hs-CRP, nmol/L^a^	Mean (SD)	10.55 (10.72)	21.28 (23.87)	28.02 (36.90)	28.10 (24.89)	8.81 (17.51)^d,i^	10.24 (15.42)	16.38 (21.26)	0.60
% of the subjects	<9.5 (low risk)	68.42	39.13	46.67	0	82.35	66.67	61.54	
	9.5–28.6 (moderate)	21.05	39.13	26.67	50.00	5.88	22.22	15.38	
	>28.6 (high risk)	10.53	21.74	26.67	50.00	11.76	11.11	23.08	
Renal profile									
Urea, mmol/L^a^	<8.3	4.00 (1.51)	4.07 (1.18)	3.35 (0.89)	6.55 (3.75)	2.92 (1.32)	3.24 (106)	4.28 (0.58)^h^	2.30
Creatinine, mol/L^a^	<106	83.68 (10.57)	82.00 (7.56)	79.07 (18.12)	148.50 (86.97)	74.65 (16.54)	61.11 (11.58)^c,d^	90.56 (12.48)^e^	3.10
Sodium, mmol/L^a^	136.0–145.0	140.33 (1.41)	137.74 (1.57)^c^	136.47 (2.70)^c,e^	139.00 (0.00)	138.38 (2.79)	139.50 (195)	137.78 (1.56)^c^	0.24
Potassium, nmol/L^a^	3.5–5.1	4.26 (0.38)	3.91 (0.28)^c^	3.75 (0.30)^c^	4.30 (0.14)	4.72 (0.48)^c^	4.33 (0.42)^h,i^	4.77 (0.62)^d,h,i^	0.44
Liver function tests									
Albumin, g/L^b^	35.0–52.0	45.60 (4.40)	47.40 (3.00)^c^	48.50 (5.70)^c^	43.80 (0.00)	45.90 (4.00)^i^	46.60 (5.70)	50.40 (3.10)^c^,^d^,^g^,^h^	1.90
Total protein, g/L^a^	66.0–87.0	73.98 (3.28)	77.30 (4.13)	79.21 (7.09)	71.90 (2.55)	75.40 (7.32)	78.80 (6.41)	76.41 (4.10)	1.12
ALP, U/L^b^	40.0–129.0	82.10 (20.40)	91.50 (39.00)	79.70 (47.60)	80.35 (0.00)	102.30 (38.40)	67.00 (14.60)^c,d,h^	66.20 (17.40)^c,d,h,i^	1.60
ALT, U/L^a^	<41.0	17.70 (5.94)	15.49 (6.81)	9.50 (4.90)^c,d^	12.10 (0.42)^c^	18.90 (10.44)^i^	27.18 (28.00)	22.47 (12.89)^g,i^	1.00
Direct bilirubin, pmol/L^a^	<5.1	2.32 (1.27)	3.03 (1.19)	4.03 (1.35)^c^		1.94 (0.93)^d,i^	0.96 (0.24)^c,d,h,i^	3.62 (1.23)^c,e,h^	6.40
Total bilirubin, pmol/L^a^	<21.0	9.23 (4.16)	8.28 (3.29)	11.25 (4.75)		4.51 (2.73)^c,d,i^	3.03 (1.43)^c,d,i^	10.60 (4.50)^e,h^	0.46
Ferritin, pmol/L^a^	67.4–898.8	304.16 (212.38)	138.67 (153.18)^c^	341.41 (343.90)	143.55 (82.94)	113.82 (110.64)^c^	91.50 (57.19)	340.56 (218.71)^d,e,h^	1.80
GGT, U/L^b^	<60.0	17.50 (11.80)	23.80 (34.00)	23.40 (31.80)	14.15 (0.00)	25.50 (63.30)	17.40 (36.40)	23.95 (17.30)	1.80

*SD* standard deviation, *TC* total cholesterol, *HDL-c* high density lipoprotein cholesterol, *LDL-c* low density lipoprotein cholesterol, *TG* triglyceride, *FBG* fasting plasma glucose, *HbA1c* glycated haemoglobin; hs-CRP, high-sensitive C-reactive protein; ALP, alkaline phosphatase, *ALT* alanine transaminase, *GGT* gamma-glutamyltransferase

^a^Expressed as mean and standard deviation

^b^Expressed as median and interquartile range due to skewed distribution, and the p-value was based on variance analysis Kruskal Wallis or Mann Whitney test

^c^p < 0.05 as compared to Semai

^d^p < 0.05 as compared to Bateq

^e^p < 0.05 as compared to Che Wong

^f^p < 0.05 as compared to Malays

^g^p < 0.05 as compared to Kanaq

^h^p < 0.05 as compared to Kensiu

^i^p < 0.05 as compared to Lanoh

The analyses of the biochemical results were referenced to the normal range for the Asians.

### Data analysis

All the Orang Asli and the Malays who were successfully recruited and provided consent to blood sampling and biochemical analysis were included in the analysis and none were excluded. Data analysis was conducted using IBM SPSS Statistics, Version 20.0 (USA). Categorical data was presented as numbers and frequencies. Continuous data with normal distribution was presented as mean and standard deviation, whereas skewed distributed data was presented as median and interquartile range.

In all cases, statistical significances were set at p value <0.05. Effects of relocation programme and gender on the biochemical tests were studied. The subjects were categorized into three groups which are Orang Asli who live in remote areas or inlands (Semai, Bateq and Lanoh), those who have been relocated to the peripheries of towns (Che Wong, Kanaq and Kensiu) as well as healthy unrelated young urban Malays with city life styles. Comparison of the biochemical profiles between the groups of Orang Asli and the Malays were analyzed using One-Way ANOVA Tukey’s HSD or Games Howell post-hoc test.

### Framingham risk score

Framingham Risk Score (FRS) is a risk prediction model which have been used in the general population to forecast cardiovascular events (CVE) and to tailor preventative therapy. It is used in this study due to its simplicity and accessibility to predict cardiovascular risks of the Orang Asli. The subjects were included in this analysis if they fulfil the inclusion criteria of the Framingham online calculator (Framingham Risk Score online) [21] to ensure the validity of the model. The validity and utility of the model was previously assessed in Malaysia. The FRS was reported to show good agreement in risk stratification and good discrimination for cardiovascular mortality with an area under the ROC curve (AUC) of 0.768 [22]. We therefore adopted the model to identify the risk and threat of cardiovascular diseases among the Orang Asli in this study. Framingham Risk Scores (FRS) were calculated for subjects who are more than 30 years and the parameters used included age, gender, total cholesterol and HDL-c level, systolic blood pressure, smoking status, as well as the history of diabetes mellitus and hypertension. Based on the Framingham Risk Score, the risks of cardiovascular diseases are divided into three categories; high (>20 %), intermediate (10 %–19 %) and low (<10 %) in 10 to 30 years to come. Only 99 subjects with their age above 30 years old, total cholesterol more than 3.6 mmol/L as well as HDL-c level above 0.8 mmol/L were included in the calculation.

## Results

The demographic characteristics of the subjects are summarised as in Table 1.

With reference to the lipid profiles (Table 2), the mean values of total cholesterol levels among the male Orang Asli were lower than the urban Malays. However, among the Orang Asli who stay in the inland, the Lanoh had the highest mean total cholesterol (TC) level with 47 % of the them having a total cholesterol level more than 5.2 mmol/L compared to less than 6 % among the Bateq and Semai. Che Wong on the other hand had the highest percentage (56 %) of TC level among the Orang Asli living at the periphery. The mean TG levels for all the sub-tribes were unremarkable, some sub-tribes showed a high percentage of TG levels above 1.7 mmol/L. For example, more than one third of the Lanoh and Che Wong had high TG levels. Whilst for the LDL levels, among the inlanders, 20 % of the Lanoh males recorded high risk and none was detected in Semai or Bateq. A higher percentage of Che Wong (22 %) was categorised as high risk among the Orang Asli at the periphery. One third of the healthy unrelated Malays recorded high level of TG. High percentage of the Orang Asli had significant lower HDL-c levels (less than 1 mmol/L), regardless of their locations. All the male Kanaq and more than 50 % of the Che Wong have HDL-c less than 1 while among the inlanders, 22 % of the Bateq, 32 % of the Semai and 40 % of the Lanoh have low HDL-c. It was interesting to observe that none of the urban Malay males have low HDL-c.

Table 2 summarizes the biochemical profiles for the males while Table 3 shows the profiles for the females. Similar with the males, the female Orang Asli also have lower total cholesterol levels when compared to the female urban Malays (Table 3). The total cholesterol levels for the inlands and peripheries female Orang Asli were 4.50 mmol/L and 4.83 mmol/L, respectively; while the urban Malays showed a higher total cholesterol level (5.20 mmol/L). A higher percentage of the female Che Wong (47 %) had TC levels of more than 5. 20 mmol/L followed by Lanoh (45 %) and Kanaq (33 %) (Table 3). As for the LDL levels, more than 30 % of the Kanaq recorded LDL levels categorised as high risk. Che Wong has the second largest percentage of individuals (21 %) with LDL level categorised as very high risk. Generally, the Orang Asli recorded a high percentage of individuals with significant lower HDL-c levels (less than 1 mmol/L). About 67 % of the female Kanaq and more than 50 % of the Kensiu have HDL-c less than 1 mmol/L; while among the inlanders, more than 16 % of the Semai have low HDL-c. It was interesting to observe that about 1 % of the urban female Malays have low HDL-c.

**Table 3:** Comparison of biochemical profiles among the female Orang Asli and female Urban Malays

Biochemical profiles	Asian Reference range	Orang Asli (female)							CV
		Inland			Periphery				
		Semai (*n* = 24)	Bateq (*n* = 4)	Lanoh (*n* = 11)	Kanaq (*n* = 9)	Kensiu (*n* = 39)	Che Wong (*n* = 19)	Malay (*n* = 89)	
Lipid profile									
TC, mmol/L^a^	Mean (SD)	4.35 (0.92)	4.35 (0.71)	4.90 (1.22)	4.91 (0.69)	4.60 (0.98)	5.28 (1.37)^c^	5.20 (0.85)^c,h^	0.81
% of the subjects	>5.2	25.00	0	45.45	33.33	25.64	47.37	49.44	
HDL-C, mmol/L^b^	Mean (SD)	1.10 (0.40)	1.45 (0.10)^c^	1.20 (0.60)	0.90 (0.30)^c,d^	0.90 (0.20)^c,d^	1.20 (0.60)^g,h^	1.70 (0.50)^c,e,g,h,i^	1.13
% of the subjects	<1.0	16.67	0	36.36	66.67	51.28	31.58	1.12	
LDL-C, mmol/L^a^	Mean (SD)	2.77 (0.74)	2.58 (0.53)	3.16 (1.01)	3.38 (0.62)	3.09 (0.93)	3.55 (1.05)	3.15 (0.78)	1.10
% of the subjects	>4.1 (very high risk)	4.17	0	18.18	33.33	10.26	21.05	14.77	
	<4.1 (high risk)	16.67	0	27.27	11.11	33.33	31.58	20.45	
	<3.4 (moderate)	45.83	50.00	36.36	55.56	17.95	31.58	43.18	
	<2.6 (low risk)	33.33	50.00	18.18	0	38.46	15.79	21.59	
TG, mmol/L^b^	Mean (SD)	0.85 (0.80)	0.60 (0.50)	1.00 (0.80)	1.40 (0.50)^c,d^	0.90 (0.70)^g^	0.80 (0.50)^g^	0.70 (0.40)^g,h^	1.60
% of the subjects	>1.7	16.67	0	18.18	11.11	10.26	15.79	3.37	
Endocrine tests									
FBG, mmol/L^b^	Mean (SD)	4.80 (0.70)	5.95 (0.40)	5.40 (1.10)^c,d^	4.90 (0.50)^c,i^	4.90 (1.20/	4.90 (1.30)^c,h^	4.90 (0.50)^c,h,i^	0.38
% of the subjects	3.5–6.0 (normal)	91.67	50.00	72.72	62.50	83.33	94.74	100.00	
	6.1–6.9 (IFG)	8.33	50.00	27.27	25.00	5.56	5.26	0	
	>7.0 (DM)	0	0	0	12.50	11.11	0	0	
HbAlc, %^a^	Mean (SD)	5.05 (0.31)	5.03 (0.47)	5.33 (0.29)	-	5.43 (0.30)^c^	5.16 (0.21)^h^	5.35 (0.24)^c^	0.80
% of the subjects	<4.8	17.39	33.33	0	-	0	0	0	
	4.8–5.9	82.61	66.67	90.91	-	94.44	100.00	98.86	
	>5.9	0	0	9.09	-	5.55	0	1.14	
Insulin, pmol/L^b^	Mean (SD)	52.40 (85.00)	62.30 (82.30)	133.80 (157.80)^c,d^	19.90 (33.80)c,i	47.2 (76.00)^g,i^	169.30 (368.30)^c,g,h^	47.10 (34.50)^e,g,i^	1.70
% of the subjects	<17.8	0	25.00	0	22.22	20.51	0	3.37	
	17.8–173.0	91.67	75.00	63.64	66.67	10.53	52.63	94.38	
	>173.0	8.33	0	36.36	0	72.22	47.37	0	
hs-CRP, nmol/L^b^	Mean (SD)	8.70 (19.30)	27.60 (44.30)	32.40 (42.90)	16.2 (98.10)	5.00 (19.30)^c^	9.40 (37.20)	5.70 (10.00)^c,i^	0.60
% of the subjects	<9.5 (low risk)	54.17	50.00	36.36	33.33	17.95	52.63	15.73	
	9.5–28.6 (moderate)	33.33	0	9.09	22.22	64.10	15.79	70.79	
	>28.6 (high risk)	12.50	50.00	54.55	44.44	17.95	31.58	13.48	
Renal profile									
Urea, mmol/L^a^	<8.3	3.25 (0.88)	3.93 (0.74)	3.47 (1.72)	3.09 (105)	2.82 (0.96)	3.32 (0.67)	3.25 (0.80)	2.30
Creatinine,	<80	58.92	66.00	62.91	83.78(12.4	58.87(11.4	71.68(14.94	57.42(7.20)^e,g^	3.10
micromol/L^a^		(8.97)	(19.51)	(21.48)	3)^c^	1)^g^	)		
Sodium, mmol/L^b^	136.0–145.0	139.95 (2.60)	140.00(8.80)	138.00 (3.00)^c^	138.00 (2.50)^c^	142.00 (8.60)^i^	140.70 (3.50)^g,i^	137.00 (2.00)^c,e,g,h^	0.24
Potassium nmol/L^a^	3.5–5.1	4.00 (0.25)	3.53 (0.57)	3.53 (0.21)^c^	4.10 (0.17)^i^	3.78 (0.47)^g^	4.13 (0.35)^h,i^	4.69 (0.45)^c,e,g,h,i^	0.44
Liver function tests									
Albumin, g/L^b^	35.0–52.0	44.45 (2.90)	45.90 (3.20)^c^	46.20 (4.70)^c^	46.20 (2.90)	44.50 (3.00)^d,g,i^	48.90 (3.60)^c,g,h^	49.20 (3.70)^g,h,i^	1.90
Total protein, g/L^b^	66.0–87.0	73.45 (5.40)	77.45 (56.90)	81.70 (4.20)^c,d^	77.80 (3.80)^c,i^	75.20 (5.30)^i^	79.10 (6.50)^c,h^	77.40 (6.70)^c,h,i^	1.12
ALP, U/L^a^	35.0–104.0	79.76 (19.00)	92.55 (26.00)	89.80 (22.14)	76.86 (16.55)	80.06 (27.82)	73.73 (26.31)	60.18 (16.41)^c^’^dhi^	1.60
ALT, U/L^a^	<33.0	16.60 (7.17)	14.98 (14.82)	13.59 (21.12)^c^	9.17 (6.61)	14.25 (9.38)	27.31 (28.83)^g,i^	11.80 (7.74)^c,e^	1.00
Direct bilirubin, pmol/L^b^	<5.1	2.00 (110)	2.20 (2.20)	2.80 (2.20)^c^	-	0.80 (1.00)^c,d,i^	0.90 (0.40)^c,d,i^	2.40 (1.20)^c,e,h^	6.40
Total bilirubin, pmol/L^a^	<21.0	8.49 (3.30)	6.90 (2.95)	9.51 (5.52)	-	3.39 (2.15)^c,i^	4.43 (1.46)^c,h,i^	7.04 (2.91)^c,h,i^	0.46
GGT, U/L^b^	<40.0	14.45 (13.90)	23.70 (52.80)	15.80 (25.10)	8.20 (8..80)^c^	20.90 (25.70)^g^	17.40 (20.60)^g^	10.30 (6.10)^c,d,e,h^	1.80
Ferritin, pmol/L^b^	29.2–337.1	167.20 (149.50)	35.05 (84.60)	86.70 (152.60)^c,d^	50.80 (145.10)^c,i^	54.40 (114.70)^g,i^	40.90 (46.10)^c,g,h^	96.00 (118.60)^e,g,i^	1.80

*SD* standard deviation, *TC* total cholesterol, *HDL-c* high density lipoprotein cholesterol, *LDL-c* low density lipoprotein cholesterol, *TG* triglyceride, *FBG* fasting plasma glucose, *HbAlc* glycated haemoglobin, *hs-CRP* high-sensitive C-reactive protein, *ALP* alkaline phosphatase, *ALT* alanine transaminase, *GGT* gamma-glutamyltransferase

^a^Expressed as mean and standard deviation

^b^Expressed as median and interquartile range due to skewed distribution, and the p-value was based on variance analysis Kruskal Wallis or Mann Whitney test

^c^p < 0.05 as compared to Semai

^d^p < 0.05 as compared to Bateq

^e^p < 0.05 as compared to Che Wong

^f^p < 0.05 as compared to Malays

^g^p < 0.05 as compared to Kanaq

^h^p < 0.05 as compared to Kensiu

^i^p < 0.05 as compared to Lanoh

The other remarkable finding is the presence of hyperinsulinemia and high hs-CRP levels among all the studied subjects. High insulin levels (>173 pmol/L) were observed in the male Semai (16 %), Bateq (26 %), Lanoh (20 %), Kensiu (12 %) and Che Wong (44 %). More than 36 % of the female Lanoh and 47 % of the female Che Wong have high insulin levels. High hs-CRP values (>28.6 nmol/L) were observed in all the Orang Asli and the Malays (male 23 %; female 13 %). Among the inlanders, Lanoh have the highest percentage of male (27 %) with high hs-CRP of more than 28.6 nmol/L. In contrast to male, more female Orang Asli have high hs-CRP levels, notably more than 50 % in Bateq and Lanoh.

None of the urban Malay male and female have mean fasting plasma glucose (FBG) that was high to denote diabetes. High FBG was observed in 4.55 % of the male Bateq, 29 % of the male Lanoh and 27 % of the male Kensiu. Only 14 % of the male Lanoh showed high HbA1c (>5.9 %). While among the female Orang Asli, about 12 % of the Kanaq and 11 % of Kensiu have high FBG of ≥7.0 mmol/L. High HbA1c was also observed in 9 % of the female Lanoh and 6 % of the female Kensiu; while 1 % of the urban females have levels of more than 5.9 %. Taken together, the Lanoh (both males and females) have higher risk for diabetes mellitus which may be associated with insulin resistance (in view of the high insulin and hs-CRP levels).

A few more biochemistry analysis showed significant differences between the male subjects of the two groups of Orang Asli and urban Malays (p < 0.001) They are the ferritin, creatinine, albumin, alkaline phosphatase, alanine transaminase, direct bilirubin as well as total bilirubin.

With reference to the Framingham Risk Scores (Table 4), 42 % of the male Orang Asli in inland have high (21 %) and moderate (21 %) cardiovascular risk scores as compared to 16.7 % for each risk scores for the male Orang Asli at the periphery. None of the urban Malay male showed high or intermediate risk of cardiovascular development in the next 10 years. However, among the females, only the inlanders showed high risk score (4.2 %) for cardiovascular diseases. Cardiovascular risks were estimated to be low and moderate for the rest of the inland Orang Asli. The urban female Malays were also found to have risk for CVDs, even though it was of low risk.

**Table 4:** Framingham Risk Score of the Orang Asli and healthy unrelated Malays

Framingham Risk Score, FRS (%)						
	Male			Female		
	Inland (*n* = 19)	Periphery (*n* = 6)	Urban (*n* = 10)	Inland (*n* = 24)	Periphery (*n* = 20)	Urban (*n* = 20)
Low risk	58.0	66.6	100	79.2	75.0	100
Moderate risk	21.0	16.7	-	16.6	25.0	-
High risk	21.0	16.7	-	4.2	-	-

## Discussion

The Government of Malaysia has put up continuous effort to bring modernisation to the Orang Asli so as to incorporate them into ‘the mainstream of the Malaysian society’. Despite this effort, majority of the Orang Asli are still having a low standard in their quality of life and the population of some subtribes remain very small in numbers. Based on our observations during the field work, the Orang Asli live in a low socio-economic status; they are lag behind in terms of education, personal hygiene and overall academic performance. Moreover, they stay in an environment which is poorly kept in terms of hygiene and resulted in a wide range of health problems such as infections. The Orang Asli at the peripheries have adopted city life with minimum physical activities and therefore a sedentary life [23]. Resettlement of the Orang Asli villages to the peripheries of towns had resulted in economic and physical distress in some of the Orang Asli [24] while the inlanders were believed to preserve living close to nature. Therefore, the Orang Asli in the inlands are believed to be healthier than those living at the peripheries of towns

As the biochemical results are gender specific, the subjects were categorized accordingly to sex before interpretation was made. The male Orang Asli showed better control of the total cholesterol levels (inland and periphery; 4.32 ± 1.03 and 4.64 ± 1.03 mmol/L respectively) when compared to the male subjects among the healthy urban Malays (5.59 ± 0.88 mmol/L). The difference was statistically significant (p < 0.001). This result is in accordance with a previous study [25] in which there were significant differences in the total cholesterol levels of the Orang Asli from deep forest hunter gatherers and resettled communities. The total cholesterol levels of the male Orang Asli were also lower than the female Orang Asli. This observation could be due to the more active life-style of the male Orang Asli as they had to earn a living for the family as compared to the females who stay at home and lead a sedentary life. This is in accordance to another study in which the body fat percentage (BFP) in the Orang Asli population was higher in women than in men and was significantly associated with age and bone mass index (BMI) [26].

Jinam et al. [27] had previously reported that Jehai and Kensiu had lower levels of cholesterol and LDL-c, but none was reported on the Che Wong and Kanaq. According to Wai et. al [28], the mean TC values for females were higher than the males and the percentage of females with ‘high risk’ scores was twice that of the males in the respective functional groups. However, in our study, only the females in Semai and Kanaq have higher TC compared to the males. And worse LDL levels and HDL-c levels were also seen among the females compared to the males Orang Asli. In Semai and Kanaq, the males usually walk a few miles to get food inside the jungle or fishing in the sea while the females stay in house and lead a sedentary life. This is further depicted with a higher TC levels among the females Orang Asli (4.83 ± 1.10 mmol/L) at the periphery compared to those staying inland (4.50 ± 1.00 mmol/L). The highest levels were seen among the urban Malay females (5.20 ± 0.85 mmol/L). The trends observed could be due to modernisation that had changed their diets and eating habits. The consumption of fast food among the Orang Asli either in the inlands or at the peripheries have contributed to the increased risk of diseases such as heart diseases, hypertension and diabetes mellitus. Orang Asli who are inlanders also have higher risks of cardiovascular diseases in the next 10 years based on the Framingham Risk Score. It is used to predict a 10-year risk of coronary heart events, including mortality due to coronary heart disease and fatal myocardial infarction (MI) by considering the presence or absence of important risk factors [29]. This was not observed 1972 by the Burns-Cox et al. whereby only the Orang Asli staying at the peripheries of the towns had adopted Western habits and had higher serum lipids than those who were living in the inlands [30].

In addition, the fasting plasma glucose levels in the Orang Asli were higher both in the males and females as compared to the urban Malays. A high percentage of the Orang Asli displayed hyperinsulineamia and high hs-CRP levels whereby these findings were associated with insulin resistance and depressed cardiovascular autonomic function in Japanese patients with type 2 diabetes mellitus [31]. According to our interview during the field studies, the Orang Asli had claimed that there were several cases of sudden death in the village. We wonder if the subjects had died of depressed cardiovascular functions, however no further evidence was made available.

Factors that contribute to high hs-CRP include obesity due to reduced physical activities and high consumption of fatty foods or meals rich in energy in some of the Orang Asli. Other conditions such as recent injuries, illnesses, and infections may raise the amount of hs-CRP and falsely elevate the estimated risks. Therefore, concurrent examination of an individual’s risk factors such as elevated levels of glucose, cholesterol, LDL-C and triglycerides is important before the diagnosis of CVD. We therefore calculated the CVD risk score of the Orang Asli using the Framingham Online Calculator which is one of the most widely used scoring system and had been validated by a research group in Malaysia [22, 32]. The parameters considered in calculating the risk include age, gender, total cholesterol level, HDL-c level, systolic blood pressure, smoking status as well as diabetic and hypertension history. It was interesting to note that higher percentage of the Orang Asli in inland have high (21 %) and moderate (21 %) cardiovascular risk scores as compared to the Orang Asli at the peripheries. This implied the resettlement programme by the Government is not the sole factor that leads to higher CVD risk but there may be other factors that are causing the underlying phenomena which may include the inherent genetic back ground of the Orang Asli.

As expected, the levels of ferritin in most of the studied subjects were in the normal range. This is in contrary to the previous report where Orang Asli have low ferritin levels and high prevalence of iron deficiency anaemia (IDA) [17]. The potassium levels among the Orang Asli were of lower normal limit. Caution is suggested as the Orang Asli may be more prone to hypokalaemia and health related problems such as heart diseases, high blood pressure, cancer, stroke, digestive disorders, and infertility. Adequate education or awareness programmes about nutrition for the Orang Asli communities are therefore important to help them maintain potassium levels within higher normal levels.

Alkaline phosphatase levels were higher among the Orang Asli living in the inlands than in the peripheries and the lowest levels were found among the urban Malays. This may be due to the higher physical activities among the inlanders compared to those at the peripheries or town. This may also point to us the potential of Vitamin D deficiency among the Orang Asli communities. One of the causes of high levels of serum alkaline phosphatase is osteomalacia and these levels are positively correlated with the severity of the disease. With raised alkaline phosphatase, osteomalacia should be considered in attempt to prevent vitamin D deficiency induced osteomalacia and rickets in children [33, 34]. A reversed correlation between Vitamin D and alkaline phosphatase levels had been reported [35].

Our findings showed that the Orang Asli staying inlands and at the peripheries are both at increased risk of cardiovascular diseases as modernisation allows easier access of the Orang Asli to towns and they have adopted changes in the diet and physical activities that lead to diseases related to modernisation. This is the first time a detailed biochemistry profile of the Orang Asli from different subtribes are reported. In addition, the study also compared the biochemical profiles of the Orang Asli with the Malays in order to investigate the effects of modernization. The data of this study is useful to provide basis for the policy maker or welfare organisation of the indigenous people in planning appropriate strategies to prevent and reduce morbidity and mortality related to cardiovascular diseases. However, other factors such as the inherent genetic background of the Orang Asli may contribute to the increased risks of CVS. The information would also form the basis for the scientific communities to conduct further studies on the pathogenesis of the abnormal biochemical parameters observed. Education and awareness on unhealthy diet and sedentary life styles for the Orang Asli are required as they may have misconception that eating fast food are rich lifestyles and perhaps a better quality of life. As some of the subtribes are small in population size, efforts are required to avoid these fragile groups from being further reduced and subsequently extinct.

We admit that the population size of the individual subtribes studied was small, and we could only observed trends rather than significant differences of some of the biochemical parameters studied. In addition, the convenient, snow ball approach used in sampling of the subjects in this study may result in some biased results as the sampling is not random. This method seems to be the most appropriate as recruitment of the Orang Asli was only successful by invitation by the trusted friends. With the help of the other Orang Asli, the investigators were able to approach and explained the details of the study before consent for participation were obtained from them.

## Conclusion

The changes of life styles and unhealthy diets adopted by the Orang Asli (both inland and periphery) as a result of modernisation have increased their risks for CVDs. Besides modernisation, other factors such as genetic of the Orang Asli may require investigation for the observed phenomena. Adequate education and awareness programmes on healthy life styles and diets are in dire need for the Orang Asli to help them to plan for a healthier community in order to ensure their sustainability.
